# Designing a Digital Intervention to Increase Human Milk Feeding Among Black Mothers: Qualitative Study of Acceptability and Preferences

**DOI:** 10.2196/67284

**Published:** 2025-03-19

**Authors:** Loral Patchen, Jeannette Tsuei, Donna Sherard, Patricia Moriarty, Zoe Mungai-Barris, Tony Ma, Elina Bajracharya, Katie Chang, William Douglas Evans

**Affiliations:** 1 Healthcare Delivery Research MedStar Health Research Institute Columbia, MD United States; 2 RAND Santa Monica, CA United States; 3 Changeable Silver Spring, MD United States; 4 Bloomberg School of Public Health Johns Hopkins University Baltimore, MD United States; 5 Benten Technologies Manassas, VA United States; 6 Department of Prevention and Community Health & Global Health Milken Institute School of Public Health George Washington University Washington, DC United States

**Keywords:** health equity, breastfeeding, qualitative, mobile health, black mothers, preferences, cultural tailoring, mobile phone

## Abstract

**Background:**

Breastfeeding rates among US mothers, particularly Black or African American mothers, fall short of recommended guidelines. Despite the benefits of human milk, only 24.9% of all infants receive human milk exclusively at 6 months.

**Objective:**

Our team previously explored the key content areas a mobile health intervention should address and the usability of an initial prototype of the Knowledge and Usage of Lactation using Education and Advice from Support Network (KULEA-NET), an evidence-based mobile breastfeeding app guided by preferences of Black or African American parents. This study aimed to identify the preferences and acceptability of additional features, content, and delivery methods for an expanded KULEA-NET app. Key social branding elements were defined to guide app development as a trusted adviser. The study also aimed to validate previous findings regarding approaches to supporting breastfeeding goals and cultural tailoring.

**Methods:**

We conducted a qualitative study using in-depth interviews and focus groups with potential KULEA-NET users. A health branding approach provided a theoretical framework. We recruited 24 participants across 12 interviews and 2 focus groups, each with 6 participants. The Data methods aligned with qualitative research principles and concluded once saturation was reached. Given the focus on cultural tailoring, team members who shared social identities with study participants completed data collection and coding. Two additional team members, 1 with expertise in social branding and 1 certified in lactation, participated in the thematic analysis.

**Results:**

All participants identified as Black or African American mothers, and most interview participants (7/12, 58%) engaged in exclusive breastfeeding. In total, 4 themes were recognized. First, participants identified desired content, specifying peer support, facilitated access to experts, geolocation to identify resources, and tracking functions. Second, delivery of content differentiated platforms and messaging modality. Third, functionality and features were identified as key factors, highlighting content diversity, ease of use, credibility, and interactivity. Finally, appealing aspects of messaging to shape a social brand highlighted support and affirmation, inclusivity and body positivity, maternal inspiration, maternal identity, social norms, and barriers to alignment with aspirational maternal behaviors as essential qualities. Crosscutting elements of themes included a desire to communicate with other mothers in web-based forums and internet-based or in-person support groups to help balance the ideal medical recommendations for infant feeding with the contextual realities and motivations of mothers. Participants assigned high value to personalization and emphasized a need to achieve both social and factual credibility.

**Conclusions:**

This formative research suggested additional elements for an expanded KULEA-NET app that would be beneficial and desired. The health branding approach to establish KULEA-NET as a trusted adviser is appealing and acceptable to users. Next steps include developing full app functionality that reflects these findings and then testing the updated KULEA-NET edition in a randomized controlled trial.

## Introduction

### Background

Breastfeeding has been shown to have numerous health benefits for infants and mothers, yet rates of breastfeeding among US mothers, particularly Black or African American mothers, fall short of recommended guidelines. The American Academy of Pediatrics [1], American College of Obstetricians and Gynecologists [2], and the World Health Organization [3] all recommend exclusive breastfeeding or exclusive human milk feeding (EHMF) for approximately 6 months after birth. Breastfeeding has been shown to be effective in reducing overall infant mortality, gastrointestinal tract and respiratory infections, sudden infant death syndrome, otitis media, and childhood obesity [4]. For mothers, breastfeeding has been associated with postpartum weight loss and a reduction in the risk of diabetes, cardiovascular disease, reproductive cancers, and postpartum depression [5,6].

Despite these benefits, only 24.9% of infants receive breast milk exclusively at 6 months [7]. Racial and ethnic disparities also exist in breastfeeding rates—non-Hispanic Black women are less likely to initiate breastfeeding and breastfeed for at least 6 months compared to the US national rate [8]. The American College of Obstetricians and Gynecologists emphasizes the importance of breastfeeding for underserved women due to the increased prevalence of adverse health outcomes among underserved groups, such as obesity, diabetes, and cardiovascular disease. Improving breastfeeding among Black or African American women not only improves short-term outcomes but also has the potential to reduce other long-term health disparities.

Barriers to initiation and continuation of breastfeeding can include structural, cultural, and societal factors, such as the need to return to work, lack of community support, or stigma around breastfeeding in public. For Black or African American women, reasons for low breastfeeding rates include not wanting to breastfeed, lack of breastfeeding information and education, having to return to work or school, lack of familial support, and being located in neighborhoods with hospitals that are less likely to implement practices that support breastfeeding [9]. Quintero et al [8] found that racial and ethnic disparities exist related to the role of breastfeeding information on breastfeeding initiation and duration. Black or African American women were less likely to receive information from their friends and family than other racial or ethnic groups.

Technology-based interventions have been shown to increase breastfeeding initiation and duration. A global meta-analysis of 15 randomized controlled trials found that technology-based interventions substantially increased the exclusive breastfeeding rate 6 months after delivery compared to usual care [10]. Mobile health (mHealth) technologies allow for greater access and dissemination of information at the exact time of need [11-13]. mHealth interventions have included a variety of modalities, including telephone support, televideo lactation consultations, SMS text messaging support, breastfeeding monitoring, and education. Several mHealth interventions have focused on the important role of peer support in increasing positive breastfeeding experiences, social connections, and a sense of belonging and self-efficacy [14]. Other mHealth technologies include location-based features, such as FeedFinder, a smartphone app using a GPS to support women in finding, reviewing, and sharing public breastfeeding places with others [15]. The My Baby Now app, which provides evidence-based information and support on infant feeding and active play from pregnancy to 18 months, found an increase in breastfeeding knowledge and EHMF intentions among Australian mothers. The authors also noted that mothers with lower levels of education found the app to be more helpful and rated it more highly [16].

Few studies have focused on the use of mHealth for breastfeeding in underserved populations. The Lactation Advice Through Texting Can Help trial connected low-income participants to breastfeeding peer counselors via SMS text messaging but did not find an impact on EHMF rates [17]. Notably, Lactation Advice Through Texting Can Help relied on text messages only and did not include additional features. Furthermore, the authors did not describe specific efforts to tailor messages to participants. A study of Breastfeeding Friend, an app with interactive advice, breastfeeding strategies and recommendations, educational content, and links to resources, also found no difference in breastfeeding rates among low-income, first-time mothers compared to a control app with digitized handouts. The authors found that women were receptive to receiving educational content through apps, but they also preferred in-person breastfeeding support, which could be provided through real-time chat functionality or SMS text messaging with experts [18]. While this intervention was designed with the intent to serve low-resource users and it delivered content at the fifth-grade reading level, it did not specify attention to cultural alignment or other aspects of social identity. Additional research is needed on culturally tailored mHealth interventions that address the barriers to breastfeeding of Black or African American women.

In our previous study, our team explored key content areas and usability of an initial prototype of the Knowledge and Usage of Lactation using Education and Advice from Support Network (KULEA-NET), an evidence-based mHealth breastfeeding intervention guided by the preferences of Black or African American parents [19]. Participants appreciated tips on returning to work, feed and diaper logs, information on infant growth and development, SMS text messaging with a lactation consultant, and a GPS map of breastfeeding-friendly locations. After the initial study was completed, the development team incorporated additional features based on these preliminary findings and supported by other research. Additional features included *social support* (eg, videos with relatable Black or African American women and family members being supportive of breastfeeding), *emotional support* (eg, personalized text messages, communication between the mother and her support individual, and educational entertainment and testimonial videos), *informational support* (eg, knowledge base), *instrumental support* (eg, breastfeeding-friendly locations, breastfeeding classes, and access to lactation support), and *appraisal* (eg, feeding and diaper logs with personalized feedback) [20-23].

### Objectives

The study aimed to assess the feasibility and acceptability of additional content and its delivery in the refined KULEA-NET app and to validate previous findings in supporting breastfeeding goals. The authors have adopted the more inclusive language of human milk feeding to report study findings.

## Methods

### Design

We conducted a qualitative study using in-depth interviews and focus groups to understand the preferences of potential users and assess the acceptability of KULEA-NET. A health branding approach provided a theoretical framework for this study [24]. This approach appeals to an individual’s self-interest and emphasizes the cost and benefit tradeoffs of a health-promoting behavior in the hope that the individual will adopt that behavior [25]. We conceptualized the KULEA-NET brand to represent mental schema that help new Black or African American mothers encompass the benefits of breastfeeding. We designed the app to offer trustworthy advice and engaging social support, which would lead to adoption and continued use. This schema captures the benefit that outweighs the costs of breastfeeding and helps new mothers succeed in consistent breastfeeding. This approach was based on the work of Evans et al [26] and others that suggest “[h]ealth branding determines behavioral choice by building consumer relationships and identification with health behaviors and their benefits.” As the nature of this study was exploratory, we used qualitative methods to gain insight into content and features that would appeal to potential users, preferred content delivery methods, and feedback on app content and prototypes. The combination of in-depth interviews and focus groups provided not only depth and breadth on our study topics but also identification of individual and contextual circumstances [27] surrounding breastfeeding.

### Ethical Considerations

This study was reviewed by the Institutional Review Board of the MedStar Health Research Institute and approved as human participant research (IRB#00005929) on March 9, 2023. Informed consent was obtained from all study participants before data collection. All study data are deidentified, and no participant identifiers are included. Study participants were compensated with an electronic US $50 gift card for the in-depth interviews and an electronic US $75 gift card for the focus groups.

### Setting and Relevant Context

This study was conducted in the United States at an economically and culturally diverse urban hospital with an academic research center and medical training programs. The hospital supports the largest number of births among all health systems in the city, >3000 births in 2022. The hospital primarily serves patients from its surrounding urban community, which was predominately White (36.3%) and Black or African American (43.5%) in 2022 [28]. In the United States, substantial racial or ethnic disparities exist in breastfeeding initiation rates. Compared to the national average, non-Hispanic Black mothers are less likely to initiate breastfeeding (73.6% vs 84.1%) [29]. In terms of resources, the Center for Disease Control and Prevention’s 2022 Breastfeeding Report Card rated this urban area as having enacted paid family and medical leave legislation and having state licensing regulations for childcare centers that were fully aligned with the standard of encouraging and supporting breastfeeding by planning for mothers to feed on-site [7]. Despite these positive indicators, other barriers to breastfeeding remain, such as breastfeeding education, familial and social support, intergenerational and historical trauma, and misinformation [8].

### Recruitment Procedures

Participants were invited to participate in the study using both active and passive recruitment approaches. A study research coordinator for women’s health studies approached patients of ambulatory health care offices specializing in obstetrics and gynecology regarding the opportunity to participate in ongoing research initiatives. This location provides a comprehensive maternal-infant program that includes a full scope of services from health care to legal, behavioral health, and social services [30]. If interested in this study, eligibility was established and enrollment completed as appropriate. In addition, lactation educators were aware of the study and referred interested patients to the study research coordinator, who determined interest, eligibility, and completed enrollment as appropriate. Finally, information about the study was shared electronically with patients through a pregnancy and postpartum care app provided to them by the health care setting. Interested individuals contacted the study research coordinator directly regarding eligibility and enrollment.

### Sample

For both in-depth interviews and focus groups, our desired population was Black or African American parents aged between 18 and 34 years who were English speaking, intended to continue breastfeeding after hospital discharge, owned a smartphone with internet access, and were willing to provide verbal informed consent. We excluded parents of infants with conditions that required admission to the neonatal intensive care unit or interfered with breastfeeding (specific conditions are listed in Textbox 1).

**Exclusion criteria for this qualitative study on support for successful human milk feeding among Black or African American mothers, highlighting medical contraindications to breastfeeding.**

**Study exclusion criteria**

- Infants born with cleft lip or palate, congenital heart defects, Down syndrome, neural tube defects, or other conditions that require admission to a neonatal intensive care unit or interfere with breastfeeding
- HIV
- Untreated, active tuberculosis
- T-cell lymphotropic virus type I or type II
- Illicit drug use
- Receiving radiation therapy
- Exposed to anthrax
- Undergone breast surgery
- Active hepatitis B or C
- Prescription drug use incompatible with lactation

We used a purposive criterion sampling method to recruit eligible patients who did not have contraindications for breastfeeding. Potential participants were contacted by the study team during a prenatal or postpartum appointment. Recruitment flyers were also posted at the center to recruit participants for the focus groups and electronically delivered to patients through a push notification associated with a remote blood pressure monitoring app used by patients receiving care at this center. In addition, snowball sampling was used to identify other potential participants that met the target population criteria. Participants were compensated with an electronic US $50 gift card for the in-depth interviews and a US $75 gift card for the focus groups.

We aimed to recruit 30 participants across the interviews and focus groups based on previous literature that recommends at least 12 participants to reach thematic saturation [31,32]. Recruitment continued until data saturation was reached on nearly all topics and no new themes emerged. In total, we recruited 24 participants across 12 in-depth interviews and 2 focus groups with 6 participants each. Due to this research’s exploratory nature and the relatively homogenous nature of our target population, our sample size provided considerable data and was found to be adequate following qualitative research principles [33].

### Data Collection

All in-depth interviews and focus groups took place on the internet via Microsoft Teams from June 1, 2023, to March 26, 2024. Recruitment occurred between June 1, 2023, and March 25, 2024. All recordings were transcribed and deleted once transcription was completed. Confidentiality was protected by assigning each participant an unidentifiable code and reporting only aggregated sample information and anonymized quotations. The study team consisted of the principal investigators (ie, multiple-principal investigator team), coinvestigators, app development technologist, study coordinator, research assistant, research nurse, and the community leadership council. Interviews lasted between 30 minutes and 1 hour, while focus groups lasted 1 hour. Team members who shared racially concordant social identities conducted data collection.

Verbal consent was obtained from every participant at the start of the interview or focus group. Before the interview or focus group, the study team provided participants with the opportunity to have any questions answered before obtaining consent. We ensured the consenting process occurred in a private location selected by the participant where other people were not present and the study team’s private office. It was recorded via Microsoft Teams.

A semi-structured interview guide and discussion guide were used for the interviews and focus groups, respectively. For the interviews, topics focused on media and information sources used during pregnancy and motherhood and potential app content and features. The topics of the focus groups centered on identity as a mother, preferences for receiving support, and desired content and features for the app.

### Data Analysis

#### Overview

The study team used deductive thematic analysis [34] to identify and interpret patterns or themes in the interview and focus group transcripts. The primary goal of the analysis was to identify themes and use them to inform the development of the content and functionality of KULEA-NET. The research team reviewed each transcript and developed a list of initial codes relevant to the research questions using the topics from the discussion guides as a starting point. An iterative process was used where codes were compared and discussed at regular team meetings during data collection. The coding dictionary was developed with each code having a distinct and mutually exclusive definition. Two team members coded the transcripts, compared codes, discussed any discrepancies, and modified the codes after obtaining agreement. To develop the themes, 2 additional research team members collaborated with those who coded the data, reviewing the codes and grouping them into meaning or a pattern that answered the research questions. A summary of the thematic analysis is presented in Table 1. All coding and analysis were performed by the coders, and no data analysis software was used.

**Table 1:** Thematic analysis of responses from Black or African American mothers who participated in focus groups and individual in-depth interviews identified 4 themes: desired content, preferred modes of content delivery, requested functionality and features, and appealing aspects of messaging to shape a social brand for the app.

Theme, theme definition, and category				Category definition
**Desired content and features**				
	**Resources, information, and features participants sought and found helpful**			
		Peer support	Connection and communication with other mothers	
		Policy and advocacy support	Knowledge of mother’s legal rights and guidance on tools to advocate for oneself	
		Access to experts	Access to lactation consultants, doulas, or health care providers through the app	
		Location-based resources	Geolocation functionality to identify helpful resources	
		Tracking and planning	Documenting the progress of pregnancy or breastfeeding and planning for exclusive breastfeeding	
**Content delivery**				
	**Method in which content is delivered to users**			
		Device type	Preferred devices or platforms	
		Messaging	Preferred messaging modality—text messaging versus notifications	
**Desired app characteristics**				
	**Preferences, requirements, or functionality participants requested**			
		Content diversity	Variety and range of content presented to app users	
		Ease of use	Capacity of an app to be used easily and effectively	
		Credibility	Trustworthy, reliable, and accurate information or sources in the app	
		Interactivity	Interaction between the user and the app that allows for real-time engagement, user input, and responsive feedback	
**Messaging**				
	**Communication style, tone, and content**			
		Support and affirmation	Emotional support, tangible support, or validation from partners, family, peers, and community	
		Inclusivity and body positivity	Acceptance of all feeding modalities, breastfeeding decisions, and comfort with the body in relation to breastfeeding	
		Maternal inspiration	Positive qualities and characteristics related to motherhood that women aim to embody	
		Maternal identity	The significance mothers attribute to motherhood and how they define their role as mothers	
		Social norms	Perceptions and attitudes on breastfeeding among family, friends, or community	
		Barriers to alignment with aspirational maternal behaviors	Challenges to fulfilling maternal behaviors that are part of a maternal identity	

#### Reflexivity

Our team participated in a reflexive process by taking notes throughout coding and consideration of thematic analysis. We used these to guide reflection on experiences related to infant feeding and considered how lived experiences informed coding and interpretation. Given the focus on cultural alignment, team members with racially concordant social identities completed data collection, coding, as well as thematic analyses related to cultural tailoring. They reflected on how their own support systems and cultural diversity within racial and ethnic communities are connected to the process of information gathering and interpretation. Team members with social branding and lactation expertise joined analysis of social branding and clinical themes only. As a group, we considered how to share these narratives with authenticity and sensitivity. A debriefing of coding and analysis was conducted with the study’s community leadership group to critically assess findings and interpretations. This review assisted the research team with identifying potential biases and validating interpretations.

## Results

### Characteristics of the Sample

Characteristics of the participants (N=24) are detailed in Table 2. Fewer identifying details were asked of focus group participants, given the group setting.

**Table 2:** All study participants identified as Black or African American women. Details of breastfeeding history were asked during individual interviews, and approximately one-third shared a history of practicing only human milk feeding with their infants.

Sample characteristics					Interviews (n=12), n (%)			Focus groups (n=12), n (%)
Identified as Black					12 (100)			12 (100)
Identified as women					12 (100)			12 (100)
**Pregnancy status**								
	Currently pregnant			6 (50)			0 (0)	
	**Not currently pregnant**			6 (50)			12 (100)	
		Primiparous	2 (33)			N/A^a^		
		Multiparous	4 (67)			N/A		
**EHMF^b^**					7 (58)			N/A
	Did not practice EHMF with first child but started EHMF with recent child			4 (36)			N/A	
	Currently, EHMF			2 (18)			N/A	
	Plan to EHMF			5 (45)			N/A	
	Has EHMF for >6 months			4 (36)			N/A	
	Attempted EHMF in past but unsuccessful			3 (25)			N/A	

^a^N/A: not applicable.

^b^EHMF: exclusive human milk feeding. Study participants were able to select more than one option, percents do not sum to 100%.

### Themes

Among the 12 interviews and 2 focus groups, four themes emerged that were related to KULEA-NET: (1) desired content and features, (2) content delivery, (3) desired characteristics, and (4) messaging.

#### Desired Content and Features

##### Peer Support

Participants described a desire to communicate with other mothers in web-based forums and internet-based or in-person support groups. They wanted to interact with mothers at different stages, such as first-time and more experienced moms, to learn about their experiences and ask questions:

I like seeing, you know, like a thread, a question and people giving their opinion their advice, their tips, how they dealt with that similar situation.

Participants referenced their appreciation of the peer support features of existing apps (eg, Peanut, What to Expect, and My Pregnancy) and websites (eg, Postpartum Support International and The Feeding Flock) they have used. Another participant described the benefits of community and not feeling alone:

I like group settings as well because I feel like when [...] another person has asked a question that you have then it’s almost like a sense of belonging because you don’t feel like you’re going crazy trying to figure something out. [...] You feel like a sense of like, ohh yes, we’re all in this together.

Some participants also mentioned the ability of peer support to fill in gaps in support from providers:

I feel like I’ve been gaining more information and support from like these other outside support groups more than I’ve been feeling supported by my provider, which is kind of sad.

##### Policy and Advocacy Support

Some participants described a desire to know their rights as mothers and for tools to advocate those rights to employers. Participants wanted to know the legality of breastfeeding in public, their rights for pumping and milk storage at work, and their rights for work breaks as pregnant mothers. One participant envisioned links to credible state-based resources:

So if they have an app that tells them “ok, you are pregnant, this is your right.” Not just say this is your right, give them an external link to like resources they can use if they don’t want to rub their boss the wrong way or whatever. When you know it is your legal right, you won’t have an issue with it and you will have something to back it up because you read it and you can go talk to HR.

##### Access to Experts

Among existing apps, participants expressed that they liked the ability to connect with experts, such as lactation consultants, doulas, or providers. Multiple participants cited the brief time they had with a lactation consultant in the hospital after birth as insufficient to meet their needs. Others discussed how they did not feel listened to by their providers and wanted access to expert-based information:

I didn’t get a chance to ask all of [my] questions, or if I did, they were kind of rushed or the answer wasn’t like a real answer.

Participants found it valuable to connect with lactation specialists during extended hours, such as when they are the only ones awake with the baby, via video calls or SMS text messaging. One participant mentioned her experience using the video chat feature of an existing app:

I used it like at 2:00 in the morning because I was, you know, desperate because I had an issue with my breast. But anyway, so it was helpful during the video call because the lactation consultant she was able to like, you know, see my breast and see like, what was the issue and provide like, you know, real time suggestions like what I can do in my case.

##### Location-Based Resources

Geolocation-based features that shared breastfeeding-friendly locations were well received by participants who evaluated the KULEA-NET prototypes:

I like it. Instead of trying to go on Google trying to figure out places where to go, at least you have, you know, the place pointed out to you where you can go.

Participants suggested adding women, infants, and children locations and places that offer free baby supplies to this feature:

I was gonna say locations where you can go that gives free diapers.

##### Tracking and Planning

Participants wanted the ability to track pregnancy and breastfeeding indicators (eg, size of baby, amount of milk pumped, and feeding times). In addition to tracking, planning for EHMF was a critical contributor to EHMF behavior. One participant discussed switching to EHMF with her second child due to planning:

I had looked into getting an IBCLC [International Board Certified Lactation Consultant] to help me and things like that - resources I didn’t know the first time, I was seeking them out in advance. Just to make sure I had an arsenal of things to help me so I wouldn’t quit.

Participants envisioned the app as a tool to help them create a plan, integrate it into their daily lives, and receive guidance throughout the planning process.

#### Content Delivery

##### Device Type

Preference for an app that could be accessed via a smartphone or tablet was preferred over a laptop, as participants found those easier to use when holding a baby, more lightweight, and accessible while on the go:

When you’re about to be a mom, your phone is your best friend.

##### Type, Frequency, and Choice of Messages and Notifications

Participants were divided regarding how they preferred to receive information from KULEA-NET. Some preferred texts, particularly if they came from the same number, while others preferred notifications. The appropriate amount of text messages or notifications ranged from 1 to 3 per week. Daily messages or notifications were thought to be too intrusive and overwhelming:

Too many texts can be annoying, aggressive, and spam-like.

Several participants also wanted a choice in either opting in or out of messages and notifications or selecting the frequency.

#### Desired Characteristics

In addition to the desired content and features, participants provided input on the characteristics KULEA-NET should exhibit.

##### Content Diversity

While KULEA-NET is designed for Black or African American women, participants recommended that the app approach cultural specificity with sensitivity to avoid the potential othering of social identities:

Because I feel like within the media, it’s always portrayed as though, umm, it’s more of a Caucasian thing to breastfeed, and maybe it’s more popular, so to speak, in that culture, I guess, but I feel like there’s plenty of black women that breastfeed celebrities that breastfeed.

Participants suggest materials include images with a diverse range of women and not only Black and White women:

I like it overall, I’m but one thing I would mention is it needs to be more diversity.

Moreover, participants did not want an app that only addressed breastfeeding but rather provided support throughout the prenatal to postnatal phases:

We have app fatigue—we need one that can transition through different stages of motherhood.

One participant discussed how a comprehensive app would reduce app fatigue:

So it can also be like a lot to continuously hop from app to app to app and so the more information that you can have in one place I think is the better. the more that you can get in one app the better.

##### Ease of Use

Information in the app should be easy to navigate, easy to read, and presented in various formats (ie, text, images, video, and audio) to appeal to various learning styles:

I’m just a visual person, so I like being able to see the person, it seems more real and personal.

One participant explained that information-heavy apps were overwhelming:

How do I say, sometimes there are apps with too much stuff and then it doesn’t feel like it’s helpful.

##### Credibility

Many participants commented on the need to emphasize the credibility of the content in the app by detailing sources, citations, or academic affiliations. Participants mentioned past experiences of receiving conflicting information from web-based sources, making them cautious of internet-based information:

A lot of the apps contradict themselves like “oh don’t give into your cravings” and then in the third trimester they’re like “eat whatever you want.” What am I supposed to be listening to?

Emphasis on credibility would make it easier and less time-consuming for participants to discern the validity of the information. While forums with peers were desirable, participants also expressed the need for a provider to moderate forums to reduce the potential of misinformation.

##### Interactivity

Video-based visits with lactation consultants supplemented by video examples of breastfeeding techniques were suggested. Participants wanted to share a live, visual display of the breastfeeding process, such as latching, with a lactation consultant to obtain effective support:

Phone or video will be most helpful so the consultant can see what you’re doing and give real time advice, or at least a phone, because you might be not hands free, but at least on the phone you can put on speaker and speak that way.

#### Messaging

##### Support and Affirmation

Participants appreciated empathetic support from their partners, parents, and children, which took the pressure off being a “perfect mom.” They also appreciated partners who took up responsibilities when mothers needed rest:

We [my partner and I] have expectations that we don’t set the bar too high. We know that in life there are various struggles and imperfections and he keeps me grounded when I feel like I have to be perfect as like a perfect mom and the perfect life.

Some participants sought comfort in seeing other mothers struggling in similar ways or found breastfeeding inspiration on social media. One participant described the encouraging influence social media had on her decision to exclusively breastfeed. She said that seeing other people doing it made her believe that she could do it too. Referring to her Facebook group, 1 participant shared the following:

Yeah, because honestly, that is one of the things that got me through was the other members, you know, somebody who’s been there, done that not just on uh educated level, but you know, somebody in my shoes.

Many participants emphasized that the app should have positive reinforcement and words of affirmation, rather than a punitive tone. Pregnancy affirmations delivered by the app helped prepare participants mentally:

I like the daily motivational quote. Basically saying you’re doing a great job, which we often need to hear as moms, even if we feel like we’re not doing a good job. So I think that’s great.

Participants did not want to be pressured or judged for their breastfeeding decisions and behaviors:

I don’t want it to make it seem like oh if you breastfeed, you are successful, but if you don’t breastfeed that you’re not successful....So just not to limit just to exclusive breastfeed[ing], like anyone could be successful.

One participant described that she does not want her partner’s opinions about what she is doing with her body. She considers breastfeeding a decision that she makes about her body and wants his support whether or not she breastfeeds:

I mean that’s great [information for partners], but I don’t want to hear my partner’s opinion about what I’m doing to my body. It’s not a driving factor to know how my partner feels about what I’m choosing to do.

##### Inclusivity and Body Positivity

Many participants expressed the need for the app to include messaging that was inclusive of all feeding modalities. The app should indicate respect for all infant feeding choices, as some mothers are unable to breastfeed due to medical issues:

...I don’t want it to seem like it’s very exclusive to like breastfeed[ing] I feel like it should be open to anyone because there are different, you know, different circumstances and situations in which a mother can’t provide breastfeeding, you know?

A couple of participants expressed regret or guilt for not breastfeeding and wanted to avoid those negative emotions:

I wanted to [breastfeed] because I figured it’s holistic, you know. That’s how, you know, my son would have been able to fight off like sicknesses and things. So, for me not to do it, it was kind of hurtful.

Body positivity was also found to be an important element of breastfeeding for participants. One participant explained how she feels more comfortable with her body with her second baby compared to her first because her breasts feel less “sexualized” and now symbolize a food source for her baby:

Definitely feeling more comfortable with my skin as far as I look as my boobs as nutrients you know. I look at it as milk, as food for the baby. I don’t look at it as sexualized you know.

##### Maternal Inspiration

When asked for examples of inspiration or influence, participants highlighted public figures who were able to balance their personal lives, careers, and other activities outside of motherhood:

Just how she balanced both working and taking care of three kids as well as, you know, still being a wife and still having her individual life going on.

Others expressed admiration for celebrity or influencer mothers who are physically active and prioritize nutrition as mothers:

Ciara is very much active, and she was very active during her pregnancy, so that was a lot of inspiration for me to be active throughout my pregnancy.

Single mothers in participants’ personal lives and in popular culture were seen as displaying strength and resilience:

She’s going through some things that an average person, you know that maybe somebody else would have broken down, but she continues to push through for her girls.

##### Maternal Identity

Participants highlighted providing emotional safety, a better relative childhood, and trust in their motherly instinct to be key qualities of a good, capable mother. Creating a safe space and promoting confidence were important goals:

I could be a good, safe space for the child, emotionally help them grow into being a productive member of society. But then also help them see that they’re uniquely gifted and uniquely made. And to help like, bring and call those things out so that they can feel like a confident person.

Participants had a desire to provide a better childhood for their children than their own:

You know, being mindful of choices that we’ve made or that were made for us that we didn’t necessarily agree with as we got older, you know, it takes that intentionality [and] awareness to say, you know, I’m gonna do this differently.

Focusing on their child’s needs rather than parenting norms and trusting their own “motherly instinct” sometimes over medical advice was valued:

The doctors may say one thing, right? You as a mom know what’s best for your baby.

##### Social Norms

Decisions on breastfeeding were often informed by practices among peers, family members, or a participant’s broader community. Seeing sisters, mothers, and aunts breastfeed provided examples of normative behavior that influenced participants’ breastfeeding decisions. One participant described her decision as “more so what I saw from my community.” Breastfeeding was also associated with challenges to traditional norms and the expectations of support systems:

Family members were a barrier for breastfeeding in the sense that it is almost not possible for family to see her the way they want to because of the breastfeeding...They’re used to bottle feeding with formula, so they’re like, give me your baby, go sleep overnight. Just trying to not discourage me intentionally from breastfeeding, but just in the sense that they wanted to help. So they’re like, “Why, it’s so, so difficult when you’re breastfeeding. Don’t [you] wanna try formula?” [It’s] not in a bad way. I know they were just trying to help, but that definitely was a hurdle for me.

##### Barriers to Alignment With Aspirational Maternal Behaviors

Mental health challenges, including stress and anxiety, were described as important barriers to being a “good mom.” One participant described how exhaustion can interfere with her intentions:

Mental exhaustion, like just being mentally tired and not wanting to do anything, just wanting to relax...When I may have other things that I could be doing or need to be doing.

## Discussion

### Multiplicity of Maternal Identity

Using a health branding framework, we initially hypothesized that a targeted intervention leveraging audience segmentation based on sociodemographic characteristics would encourage intended behavior change [24,25]. However, our previous study revealed mixed responses to a culturally tailored intervention featuring Black or African American imagery. Participants informed us that providing for one’s child was a universal desire that should not require race- or ethnicity-specific content [19]. In this study, we delved into participants’ perceptions of maternal identity to better inform our communication approach. Our findings reiterated commonalities in maternal identity, particularly shared motivations of fostering emotional safety and providing a better childhood. Furthermore, sources of maternal inspiration highlighted were not exclusively Black or African American and were mentioned for a variety of desirable characteristics, such as success in balancing the multiple components of mothers’ lives. Racial identity is merely one aspect of maternal identity among Black or African American mothers. Effective role models must speak to the multidimensional nature of motherhood, particularly the skillful managing of these diverse identities.

Previous research has shown the importance of avoiding the uniform treatment of sociodemographic groups, instead recognizing the multiple identities and intersecting factors that exist within them. A systematic review of mHealth apps among populations considered vulnerable, specifically low-socioeconomic status and historically marginalized racial and ethnic groups, found few improvements in health outcomes. The review underscored the need for further research that differentiates between broad categories of low-socioeconomic status and racial and ethnic minority groups to understand how mHealth tools can effectively address a range of needs [35]. Our mHealth intervention must appeal to the varied needs and lived experiences of mothers by incorporating features that consider sociocultural norms, environmental factors, household composition, resources, and availability of breastfeeding role models. KULEA-NET includes features informed by the barriers Black or African American mothers may face, such as breastfeeding education and tips, a GPS-based map of breastfeeding-friendly spaces, and feeding and diaper change logs. The delivery of this content will address the multiplicity of identities among Black or African American mothers, which includes using imagery and messaging that speaks to a range of current and aspirational identities and helps them feel represented.

### Nonoppositional Messaging

While EHMF for 6 months is recommended, our findings revealed that mothers wanted a more inclusive approach to breastfeeding—one that supports more options to accommodate the realities of a mother’s circumstances and available resources. A recent study evaluated the “breast is best” narrative and noted a similar finding where mothers sought a more nuanced view toward breastfeeding and formula feeding to recognize a diversity of lived experiences and avoid inducing feelings of shame or failure when mothers were unable to EHMF [36].

In social marketing and health branding, oppositional and nonoppositional strategies are commonly used. Nonoppositional strategies aim to promote behavior change without directly challenging existing beliefs or practices (eg, eating 5 servings of fruits and vegetables), while oppositional strategies confront or compete with unhealthy messages (eg, antitobacco or antismoking messages) [25]. Our participants discussed workplace challenges to breastfeeding and the desire to know their rights or have tools to advocate for breastfeeding at work. Although mothers may intend to exclusively breastfeed, they may lack the means to achieve this goal. Employment before 6 months without adequate breaks and space for pumping has been shown to be a key barrier to EHMF [37,38]. Asiodu et al [39] emphasized the need to discuss combination feeding openly and alleviate some of the emotional burden and stress associated with not meeting expectations or societal pressures of EHMF. The tone and messaging of KULEA-NET must take a nonoppositional approach and acknowledge the circumstances that influence the ability of women to breastfeed. It will aim to provide positive reinforcement and encouragement to support mothers on their breastfeeding journey, rather than generate guilt or shame.

### Critical Role of Support

Various forms of tangible and emotional support emerged as key features participants desired in KULEA-NET. Tangible support included peer support, partner support, access to experts, and assistance in navigating breastfeeding rights with employers. In addition, mothers expressed a strong need for emotional support to cope with the pressures of motherhood. Participants voiced the importance of nonjudgmental acceptance and encouragement of a mother’s choices.

Access to breastfeeding support has been identified as an important mediator, accounting for two-thirds of the Black-White disparity in breastfeeding outcomes [9]. Influential supportive figures include not only providers and community-based experts but also family members, partners, friends, peers, church members, and even strangers. Moreover, Black or African American women are most likely to initiate and continue breastfeeding if they receive adequate support (ie, assistance with lactation, domestic activities, childcare, and groceries) from these sources during the first 2 weeks post partum [40]. Support from these various sources can help mothers overcome barriers to EHMF.

In this study, participants discussed examples of tangible and emotional support. Intimate relationships, such as partners, mothers, aunts, and grandmothers, not only shared some of the burdens of motherhood but also served as role models and fostered a normative culture of breastfeeding. Peers allowed mothers to learn from others’ experiences, ask questions, and fill in when providers did not provide sufficient breastfeeding support. Peer support could also be internet-based, through forums or social media, in providing a sense of belonging, connection, and empowerment. Gyamfi et al [40] stressed the importance of exploring all avenues, including internet-based platforms, to establish culturally sensitive breastfeeding groups among peers in the Black or African American community to promote breastfeeding. Peer support features within an mHealth app can provide emotional support and community engagement and address a variety of unique needs in the Black or African American community.

KULEA-NET aims to integrate these findings by offering an app that recognizes the diverse lived experiences of Black or African American women, addresses both EHMF and combination feeding, and provides social support to improve breastfeeding outcomes. Future research will evaluate the efficacy of the fully developed version of this mHealth intervention in increasing breastfeeding rates through a randomized controlled trial. Findings of that trial will include usability and user engagement metrics and compare outcomes of exclusive human milk feeding success rates.

Future research should examine the conceptual model underlying KULEA-NET and its focus on cultural tailoring. This would involve evaluating the pathway of change and mediating factors, such as social support and app features, and their effect on breastfeeding outcomes. In addition, further research should explore the optimal timing for introducing the app (eg, enrolling participants in a KULEA-NET promotion study before vs after delivery) as well as the dosage and frequency of use for specific features. Studies should seek to identify the mechanisms of change through which KULEA-NET works.

### Limitations

While our qualitative methodology provided a range of positive and negative perspectives on the KULEA-NET app and detailed information about the experiences of Black or African American mothers, there are some limitations to note. Our inclusion criteria included a requirement that participants were willing or intended to breastfeed, which meant that our sample may have been predisposed to breastfeed and may not have been fully representative of Black or African American mothers. In addition, multiple participants worked in health care, and our sample was informed of the benefits of EHMF compared to other nursing methods, which limited the external validity of our findings. Another limitation was inconsistency in the interview questions asked of participants. Some new questions were introduced in later interviews that were not included in earlier interviews, mainly questions concerning participant knowledge of their rights as mothers. For these questions, we had a smaller sample size, resulting in less detail on this topic.

### Conclusions

Formative research suggests that the KULEA-NET app is acceptable and beneficial to participants. The personalization, support, and maternal identification features stood out as key benefits provided by the app. Implementation and promotion of app use represent important next steps. Future study developments will include full app functionality and testing in a randomized controlled trial.

## Appendix

Multimedia Appendix 1 COREQ checklist.

## Abbreviations

COREQ: Consolidated Criteria for Reporting Qualitative Studies
EHMF: exclusive human milk feeding
KULEA-NET: Knowledge and Usage of Lactation using Education and Advice From Support Network
mHealth: mobile health

## Data Availability

Deidentified study transcripts are available upon reasonable request to the corresponding author, with an established data transfer agreement.
